# Genome Analysis of *Listeria monocytogenes* Sequence Type 8 Strains Persisting in Salmon and Poultry Processing Environments and Comparison with Related Strains

**DOI:** 10.1371/journal.pone.0151117

**Published:** 2016-03-08

**Authors:** Annette Fagerlund, Solveig Langsrud, Bjørn C. T. Schirmer, Trond Møretrø, Even Heir

**Affiliations:** Nofima, Norwegian Institute of Food, Fisheries and Aquaculture Research, Ås, Norway; Institut Pasteur Paris, FRANCE

## Abstract

*Listeria monocytogenes* is an important foodborne pathogen responsible for the disease listeriosis, and can be found throughout the environment, in many foods and in food processing facilities. The main cause of listeriosis is consumption of food contaminated from sources in food processing environments. Persistence in food processing facilities has previously been shown for the *L*. *monocytogenes* sequence type (ST) 8 subtype. In the current study, five ST8 strains were subjected to whole-genome sequencing and compared with five additionally available ST8 genomes, allowing comparison of strains from salmon, poultry and cheese industry, in addition to a human clinical isolate. Genome-wide analysis of single-nucleotide polymorphisms (SNPs) confirmed that almost identical strains were detected in a Danish salmon processing plant in 1996 and in a Norwegian salmon processing plant in 2001 and 2011. Furthermore, we show that *L*. *monocytogenes* ST8 was likely to have been transferred between two poultry processing plants as a result of relocation of processing equipment. The SNP data were used to infer the phylogeny of the ST8 strains, separating them into two main genetic groups. Within each group, the plasmid and prophage content was almost entirely conserved, but between groups, these sequences showed strong divergence. The accessory genome of the ST8 strains harbored genetic elements which could be involved in rendering the ST8 strains resilient to incoming mobile genetic elements. These included two restriction-modification loci, one of which was predicted to show phase variable recognition sequence specificity through site-specific domain shuffling. Analysis indicated that the ST8 strains harbor all important known *L*. *monocytogenes* virulence factors, and ST8 strains are commonly identified as the causative agents of invasive listeriosis. Therefore, the persistence of this *L*. *monocytogenes* subtype in food processing facilities poses a significant concern for food safety.

## Introduction

*Listeria monocytogenes* is an opportunistic Gram-positive pathogen responsible for the foodborne disease listeriosis. It has a saprophytic lifestyle and is ubiquitously present in the environment, including in water, soil and vegetation, and can survive in food products and in food processing plants. Contamination of food products is typically from sources in the food processing environment. The ability of *L*. *monocytogenes* to grow at low temperatures, form biofilms and persist in food processing plants has made this bacterium a significant threat for contamination of ready-to-eat (RTE) foods, posing a significant food safety challenge [1–3].

Several studies employing classical molecular subtyping methods such as pulsed-field gel electrophoresis (PFGE), multilocus sequence typing (MLST), and multiple-locus variable-number tandem-repeats analysis (MLVA) have revealed that certain molecular subtypes of *L*. *monocytogenes* are repeatedly isolated from food processing environments for extended periods of time, while other subtypes are found only sporadically [2, 3]. The term “persistence” is often used to describe the long-term survival of a pathogen in a particular habitat, e.g. in a food processing plant. The concept often implies that persistent strains harbour a phenotype or an adaptation which enable them to survive in a given environment, although no robust correlation between persistent strains and phenotypes such as biofilm formation or stress and disinfectant resistance has been detected [2–4]. Persistence is often defined as the recurrent isolation of the same molecular subtype in the same food processing plant over a long time period [2–4]. The observed persistence may be due to resident strains that have become established in niches in the plant, or be caused by frequent introduction of strains of the same subtype e.g. through raw materials or the surrounding environment. *L*. *monocytogenes* isolates recovered from a food production facility after cleaning and disinfection are more likely to represent resident strains, compared to when sampling is performed during production. It should also be noted that the ability to differentiate between a potentially resident clonal strain and repeated introduction of similar but non-clonal strains from the outside environment will depend on the discriminatory power of the subtyping method employed [2].

Whole genome sequencing (WGS) has in recent years emerged as the method of choice for epidemiological surveillance and investigation of outbreaks caused by bacterial pathogens. Whole-genome single nucleotide polymorphism (SNP) analysis is able to reveal detailed information about phylogenetic relationships at a resolution not obtainable using classical molecular subtyping methods such as PFGE and MLST [5–7]. The method has been applied to epidemiological analyses of several listeriosis outbreaks [8–11]. Isolates of the same outbreak strain show very little genetic diversity and SNP analysis is often required to differentiate strains and track their origins and transmission patterns during an outbreak. The same would apply to persistent strains of *L*. *monocytogenes* in food processing environments. Genome sequencing may be used to reveal information about the microevolution of such strains and to track the source of contamination. Genome sequencing has also been employed in attempts to discover genetic traits linked to persistence in *L*. *monocytogenes* [10, 12, 13, 14], for which the underlying molecular mechanisms are largely unknown [2–4].

*L*. *monocytogenes* of MLST sequence type 8 (ST8) was identified as persisting for over three years in a raw salmon processing plant in Denmark [15, 16], and is commonly found in food products and food processing environments [17–21]. *L*. *monocytogenes* ST8 strains belong to clonal complex 8 (CC8), and thus also to the proposed epidemic clone 5 (ECV), which was the predominant subtype of *L*. *monocytogenes* responsible for cases of human listeriosis in Canada between 1988 and 2010 [22]. Several other cases of invasive listeriosis has been reported to be caused by ST8 strains [23–26], indicating that ST8 strains have full pathogenic potential. The persistence of ST8 strains in food processing environments, where cross-contamination of finished food products may occur, is therefore a serious food safety issue. Consequently, a closer investigation of this particular subtype is of key interest.

During surveillance studies of *L*. *monocytogenes* in salmon and meat processing facilities in Norway, we repeatedly isolated *L*. *monocytogenes* of the same MLVA profile in one salmon processing facility and in two poultry processing plants. Isolates of this MLVA profile were shown to correspond to MLST ST8. We have in the current study sequenced the genomes of five of the collected *L*. *monocytogenes* ST8 stains. Three were isolated in the salmon processing facility, in which this molecular subtype of *L*. *monocytogenes* was recurrently detected over a period of 13 years. One of these strains was from 2001 and two were isolated ten years later. The two remaining sequenced strains were from two different poultry processing facilities where transmission of ST8 *L*. *monocytogenes* was suspected to have occurred through transfer of contaminated equipment from one facility to the other. Comparative genome analysis was performed with the aim to determine whether the strains are likely to represent resident clonal strains.

The genomes of the five currently sequenced strains were furthermore compared with five additional available ST8 genomes, of which one was a persistent clone isolated from a Danish salmon processing facility [15, 16], two were isolated from ricotta cheese in Italy [20], and one was a clinical strain isolated from a hospitalized patient in Shanghai, China [25]. Inclusion of these strains in the study enabled examination of ST8 strains isolated from a range of food and food processing environments (salmon, poultry, and cheese) in addition to a human clinical strain, and allowed the reconstruction of the evolutionary history of the ST8 strains using SNP data. Genetic determinants and characteristics of the ST8 genomes were analysed and discussed.

## Materials and Methods

### Bacterial strains and molecular subtyping

Five *L*. *monocytogenes* strains were sequenced for this study (Table 1). MF4245, MF3949, and MF4077 were isolated from a salmon processing plant in Norway in 2001 and 2011. Raw materials at this facility are processed farm-raised salmon (gutted with head) from Norway. MF5377 was isolated from a Norwegian poultry processing plant in 2013. MF3949, MF4077, and MF5377 were isolated from samples taken using neutralizing sampling cloths (SodiBox) according to ISO method 18593 and analysed according to ISO 11290–1 with selective enrichment in half-Fraser and Fraser broth and final plating on RAPID’*L*.*mono* agar (Bio-Rad). MF4245 was from a swab sample taken as part of the salmon processing plant’s own sampling program and analysed at a routine laboratory according to ISO methods 18593 and 11290–1. MF5369, isolated from a second Norwegian poultry processing facility in 2011, was obtained from the Norwegian Veterinary Institute as isolate VI56781. It was isolated as part of the poultry processing plant’s own sampling program and analysed at a routine laboratory. MLVA subtyping was performed according to the method described by Lindstedt *et al*. [27] at the Norwegian Institute of Public Health. MLST was performed as previously described by Ragon *et al*. [24]. Alleles and sequence types for MLST were compared with those available in the Institute Pasteur's *L*. *monocytogenes* MLST database at http://bigsdb.web.pasteur.fr/listeria/listeria.html.

**Table 1:** *L*. *monocytogenes* CC8 genome sequences used in the present study.

Strain	MLST sequence type	Source of isolation	Year of isolation	Reference	GenBank Accession number(s)
MF4245	ST8	Salmon processing facility, Norway	2001	This study	LKVA00000000
MF3949	ST8	Salmon processing facility, Norway	January 2011	This study	LKUZ00000000
MF4077	ST8	Salmon processing facility, Norway	October 2011	This study	LQXC00000000
MF5369	ST8	Poultry processing facility A, Norway	2011	This study	LKUY00000000
MF5377	ST8	Poultry processing facility B, Norway	2013	This study	LKUX00000000
R479a	ST8	Cold-smoked salmon (final product), sampled at salmon processing facility in Denmark	1996	[15, 16]	HG813247, HG813248
SHL004	ST8	Clinical isolate (human blood) from hospitalized patient in Shanghai, China	2008	[25]	APID00000000
Lm21045	ST8	unknown	unknown	Unpublished. Sequenced by DOE Joint Genome Institute	JHZQ00000000
Lm_1823	ST8	Ricotta cheese, Italy	2012	[20]	AZIU00000000
Lm_1889	ST8	Ricotta cheese, Italy	2012	[20]	AZIY00000000
08–5578	ST292	Clinical isolate (human blood), Canada	2008	[8]	CP001602/CP001603
08–5923	ST120	Clinical isolate (human blood), Canada	2008	[8]	CP001604
HPB2088	ST120	Food processing facility, Canada	1994	Unpublished. Sequenced by Health Canada	JOKU00000000
HPB5415	ST292	Beef deli slices, Canada	2008	[28]	JOKV00000000
IZSAM_Lm_14–16064	ST16	Baked ham, Italy	2014	Unpublished. Sequenced by IZSAM	LJAR00000000
Lm60	ST551	Clinical isolate (human blood), Switzerland	2006	[29]	CP009258

### DNA isolation and genome sequencing

For purification of genomic DNA, cells were lysed using Lysing Matrix B and a FastPrep instrument (both MP Biomedicals) and DNA isolated using the DNeasy Blood and Tissue Kit (Qiagen). Libraries for genome sequencing were prepared using the Nextera XT DNA Sample Preparation Kit (Illumina) and sequenced using paired-end (PE) 2 × 300 bp reads on a MiSeq instrument (Illumina). The raw reads have been deposited to the National Center for Biotechnology Information (NCBI) Sequence Read Archive (SRA) under accession numbers SRR3099222, SRR3099221, SRR3099223, SRR3099224, and SRR3099225.

### Genome assembly

As there is no single best approach to *de novo* genome assembly [30], several assembly programs were tested using iMetAMOS [31] to identify an assembly program which would perform well with our datasets. Prior to assembly, removal of adapter sequences and q15 quality trimming was performed using *fastq-mcf* from the *ea-utils* package [32]. Genome assembly was performed using SPAdes v3.0.0 [33] with the—careful option and three k-mer sizes, including the best k-mer size determined by KmerGenie [34]. Contigs with size <500 bp and with coverage <24 were removed from the assemblies. The genome assemblies were evaluated using QUAST v2.2 [35] and the sequences were annotated using the NCBI Prokaryotic Genomes Automatic Annotation Pipeline (PGAAP) server (http://www.ncbi.nlm.nih.gov/genome/annotation_prok/). The genome sequences have been deposited at DDBJ/EMBL/GenBank under the accession numbers listed in Table 1. The versions described in this paper are versions XXXX010000000.

### Public data sources

Five publicly available genome sequences of ST8 (CC8) *L*. *monocytogenes* were included in the comparative genome analysis. These were the complete sequences of the *L*. *monocytogenes* R479a chromosome and pLMR479a plasmid, which were used as reference sequences [16], and draft genomes of *L*. *monocytogenes* strains SHL004 [25], Lm_1823, Lm_1889 [20], and Lm21045 (Table 1). The following six publicly available non-ST8 CC8 *L*. *monocytogenes* genome sequences were included in the phylogenetic analysis: 08–5578 (ST292), 08–5923 (ST120) [8], Lm60 (ST551) [29], HPB5415 (ST292) [28], HPB2088 (ST120), and IZSAM_Lm_14–16064 (ST16) (Table 1). Additional genomes used for evaluation of prophage, plasmid, or accessory genomes of ST8 strains were *L*. *monocytogenes* strains 4423, 3253, Lm_1880 (all ST121/CC121) [13, 20], 10403S (ST85/CC7) and EGD-e (ST35/CC9) [36], and the plasmid of *L*. *monocytogenes* 6179 (ST121/CC121) [13]. GenBank accession numbers for these sequences are CBXR000000000, JYJO00000000, AZIZ00000000, CP002002, AL591824, and HG813250. The genome sequences were downloaded from the GenBank database (http://www.ncbi.nlm.nih.gov/).

### Analysis of sequence data

Contigs from the genome assemblies were oriented and ordered by mapping them to the sequence of the R479a chromosome and plasmid using the Mauve v2.3.1 Contig Mover [37]. Mauve was also used to identify larger indels and structural variation between genomes.

For detection of SNPs and indels, two approaches were employed: First, Illumina reads filtered using *fastq-mcf* (see above) from the five ST8 strains sequenced in the current study were mapped against the chromosome and plasmid sequences of *L*. *monocytogenes* R479a using Bowtie2 v.2.2.5 [38] and variants were called with FreeBayes v.0.9.7 [39] with options—ploidy 1,—pvar 1, and—left-align-indels. Using these settings, all called variants were present in all examined strains. The data was filtered to remove variant call positions where one or more strains displayed read depth (DP) <10 or an reference allele count (RO) >1. Second, the MUMmer [40] application Nucmer (with options—maxmatch and -c 100) was used to align the assembled genome sequences of publicly available CC8 genomes (see previous section) to the R479a chromosome and plasmid sequences. Then the MUMmer application show-snps (with -C option) was used to call SNPs and indels from the alignments. All variants with more than 20 nt distance between a variant position to the nearest mismatch in the same alignment (i.e. [BUFF]<21) were filtered out. Positions where variant calls were not in agreement between both variant callers were excluded in the final table of variants (S1 Table).

For phylogenetic analysis, all indels and all variant positions with missing data in one or more strains, including positions within the plasmid and the prophage regions of R479a (regions 1233337 to 1275517 and 2366510 to 2406159 for the prophages at the tRNA-Arg-TCT and *comK* loci, respectively) were eliminated. This final set of 872 SNPs for each genome was concatenated to a single alignment. Subsequently, a phylogenetic tree was inferred in MEGA6 [41] using the Maximum Parsimony method. The Maximum Parsimony tree was obtained using the Subtree-Pruning-Regrafting algorithm with search level 1 in which the initial trees were obtained by the random addition of sequences (10 replicates), branch lengths were calculated using the average pathway method, and bootstrap confidence values were generated using 1000 replicates. The tree was rooted on *L*. *monocytogenes* IZSAM_Lm_14–16064.

Prophage regions were identified by examination of previously identified *L*. *monocytogenes* prophage insertion sites [8, 9, 42, 43] and screening the genome sequences using the Phast phage search tool [44]. Plasmid and prophage sequences were compared using BLAST+ v2.2.30 [45]. In cases where plasmid or prophage sequences were present on more than one contig, these contigs were joined after orientation and ordering using Mauve. Where appropriate, contigs were joined by searching for short overlapping sequences between neighbouring contigs prior to BLAST comparison. Phage and plasmid genome maps were drawn using EasyFig v2.1 [46].

Search for CRISPR sequences was performed using CRISPRFinder [47]. Determination of accessory ORFs was performed using PanSeq [48] (https://lfz.corefacility.ca/panseq). Predicted protein function was assessed using the InterProScan tool (http://www.ebi.ac.uk/interpro). Alignments were created using CLC Main Workbench 7.5 (CLCbio).

## Results

### Persistence of *L*. *monocytogenes* ST8 in Norwegian food processing plants

*L*. *monocytogenes* with the MLVA profile 6-9-18-16-6 (profile corresponding to VNTR loci LMV6-LMV1-LMV2-LMV7-LMV9 [27]) were repeatedly isolated from sanitated conveyor belts and slicing machines in a Norwegian salmon processing facility during an eight-week period in 2001. During the years 2011–2014, a total number of 41 isolates with the same MLVA profile was isolated from various product contact surfaces (conveyor belts) and product non-contact surfaces (drains, floors, footwear, etc) in the same plant. Of the 41 obtained isolates, 28 were from samples taken after sanitation. When testing samples from raw fish (gutted salmon with head) obtained from 23 different suppliers of raw materials to the facility during a six month interval from September 2013 to March 2014, isolates with this MLVA profile were detected in salmon from two of the suppliers. Four of the isolates from the processing environment (two each from 2001 and 2011) were typed using MLST and belonged to ST8. Repeated isolation during a timeframe of 13 years of the same molecular subtype of *L*. *monocytogenes* in the same salmon processing facility indicated that this subtype has a good ability to persist in salmon processing environments, either in the processing facility itself or another environment that serves as a source for reintroduction.

*L*. *monocytogenes* with the same MLVA profile as the one found in the salmon processing facility (described above) was also detected in surface samples from a conveyor belt after sanitation in a Norwegian poultry processing facility during two visits in December 2012 and October 2013. Prior to installation of this conveyor belt in September 2012, *L*. *monocytogenes* with this MLVA profile had never been detected in this facility. Notably, the conveyor belt in question had been relocated from a second Norwegian poultry processing plant, in which the *L*. *monocytogenes* subtype in question had repeatedly been detected. One isolate of this MLVA profile from each of the two facilities were typed using MLST, and both were shown to belong to ST8. Repeated isolation of this molecular subtype at two facilities suggests that it has the ability to persist in poultry processing environments. The data also suggested that the contamination had been transferred from one plant to another through contaminated processing equipment.

### Genome sequencing of five *L*. *monocytogenes* ST8 strains

To examine the genetic relationship between ST8 strains collected from the Norwegian salmon processing facility, three isolates collected over a period of ten years were subjected to WGS. These were *L*. *monocytogenes* MF4245 isolated in 2001 and *L*. *monocytogenes* MF3949 and MF4077 from January and October of 2011 (Table 1). To investigate whether a *L*. *monocytogenes* contamination in a poultry processing plant could be linked to purchase of a used conveyor belt from another poultry processing plant, one ST8 isolate from each facility was examined using WGS. *L*. *monocytogenes* MF5377 was isolated in 2013 at the processing facility where the used conveyor belt was installed, while MF5369 was isolated in 2011 at the facility in which the conveyor belt originated (Table 1). Sequencing of these five strains would furthermore enable an examination of genetic determinants and characteristics of ST8 isolates persisting in food processing environments.

The main general features of all five genome assemblies are shown in Table 2. The genome size was around 3.0 Mb and the GC content 37.8% for all five genomes, which is in the range typically found in *L*. *monocytogenes* genomes. *In silico* MLST confirmed that all five sequenced strains were of the ST8 subtype.

**Table 2:** Assembly quality metrics for the sequenced *L*. *monocytogenes* genomes.

	MF4245	MF3949	MF4077	MF5369	MF5377
No. of paired-end sequences	1 552 015	345 697	1 080 312	1 370 819	1 267 740
Assembly size (bp)	3 009 817	3 009 206	3 009 599	3 008 780	3 009 089
N50	579 563	251 267	438 935	579 519	578 797
Assembled coverage (average)	113×	29×	53×	98×	118×
Contig number ^a^	14	21	14	14	16
GC content	37.83%	37.83%	37.82%	37.83%	37.82%
No. of Genes	3 035	3 034	3 033	3 032	3 031
No. of CDS	2 951	2 949	2 967	2 950	2 949
No. of pseudogenes	19	23	19	23	21
No. of rRNAs (5S, 16S, 23S)	4, 1, 3	4, 1, 2	4, 1, 2	2, 1, 1	1, 2, 3
No. of tRNAs	56	54	54	54	54

^a^ Only contigs with size >500 bp and with coverage >24 were included.

### Whole-genome SNP phylogeny for CC8 strains

For comparative genomic analysis of the sequenced strains, *L*. *monocytogenes* R479a [16] was selected as the reference strain. This strain was the only ST8 strain for which a complete genome sequence was available. It was isolated from smoked salmon in a fish processing plant in Denmark in 1996, belongs to serovar 1/2a, and represents a random amplified polymorphic DNA (RAPD) profile which was shown to persist in this facility for over two years [15, 16]. R479a harbors two prophage regions, inserted into the *comK* and tRNA-Arg^TCT^ loci, and one circular plasmid of 86 Kbp (pLMR479a).

**Fig 1:**
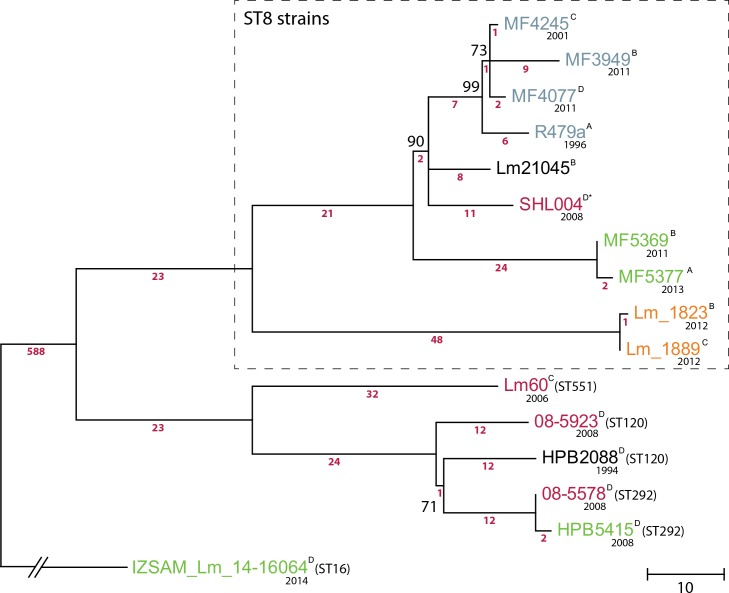
Phylogeny for the *L*. *monocytogenes* CC8 isolates based on whole-genome SNP analysis. Maximum Parsimony tree based on 872 non-homoplasic chromosomal SNPs. The tree was rooted on *L*. *monocytogenes* IZSAM_Lm_14–16064. Bootstrap support is shown for nodes with <100% bootstrap support. Branch lengths are in the units of the number of changes over the whole sequence (except for the branch for IZSAM_Lm_14–16064 which has been shortened for clarity), and the number of contributing SNPs is indicated below each branch (numbers in pink). For details on the identity of the indicated SNPs differentiating the strains, see S1 and S2 Tables. The ST8 lineage is indicated by a box with dashed line, for the other strains the ST is indicated in parenthesis after strain names. The origin of strains is indicated by the colour of the strain name: Blue; salmon processing facility, green; poultry processing facility or meat, orange; cheese, pink; human clinical strain. Superscripts after strain names indicate the variant of the *hsdRMS* locus found in each strain (see text, Fig 2 and Table 3 for details). The year of isolation (when known) is noted below the name of each isolate.

To investigate the relationships between the five sequenced ST8 strains (MF4245, MF3949, MF4077, MF5369, and MF5377), Illumina reads were mapped to the *L*. *monocytogenes* R479a chromosome and plasmid sequences. Variants with sequence coverage of more than 10 were retained. In a second approach, variants were called based on alignments of the assembled genome sequences and the R479a reference sequences obtained using the nucmer program from MUMmer. In order to place the sequenced strains in context with other CC8 strains, all 10 additional publicly available *L*. *monocytogenes* CC8 genome sequences (see Table 1) were included in the MUMmer analysis. Of these strains, four were of ST8. The results from the two analyses were filtered and compared (see Methods section). For variants detected in the five sequenced ST8 strains, only positions called by both methods were retained. Of note, three SNPs present only in strain MF3949 were detected in both approaches but filtered out due to low read coverage in the mapping based variant calling analysis. The final set of variants is reported in S1 Table. Details of the SNPs and indels detected in the ten ST8 strains (Table 1) are presented in S2 Table. For inferring the phylogeny, all indels and SNPs present in plasmid and prophage sequences were excluded, resulting in identification of 872 non-homoplasic SNPs used to draw the phylogeny of the *L*. *monocytogenes* CC8 genomes. The Maximum Parsimony tree generated by the whole-genome SNP analysis is shown in Fig 1. The analysis shows that the ten ST8 genomes included in the analysis consist of two main clades. The first clade comprises eight strains, including strains from salmon and poultry processing environments in Norway and Denmark, as well as a human clinical isolate from China. The second clade, consisting of the two strains from Italian cheese, differs from the first clade by 71 SNPs or short indels in the genome backbone (Fig 1, S1 and S2 Tables).

**Table 3:** Non-SNP differences between the *L*. *monocytogenes* ST8 genomes.

Genetic trait	Name and origin of *L*. *monocytogenes* strains									
	Salmon processing facilities				Poultry processing facility		Clinical		Cheese	
	R479a	MF4245	MF3949	MF4077	MF5369	MF5377	SHL004	Lm21045	Lm_1823	Lm_1889
Variant of the *hsdRMS* Type I restriction modification-system (LMR479A_0528–0532)^a^	A	C	B	D	B	A	D^b^	B	B	C
Copies of Cna_B domains (PF05738) in Peptidoglycan binding protein, SdrE (LMR479A_0168)	4	4	4	4	4	3	ND^c^	ND^d^	ND^d^	ND^d^
Copies of the ncRNA LhrC (RF00616) between LMR479A_0232 and LMR479A_0234	2	3	3	3	3	3	ND^d^	3	3	ND^d^
Copies of 213 bp repeat between LMR479A_1288 and LMR479A_1289 in tRNA-Arg^TCT^ prophage	3	2	2	2	1	1	1	Phage not present	Phage not present	Phage not present
Copies of C-term_anchor domains (PF13461) in internalin J (LMR479A_2522)	5	4	4	4	4	3	ND^d^	ND^d^	ND^d^	ND^d^
Presence of orthologue to gene LMR479A_p0083 on plasmid	yes	yes	yes	yes	no	yes	yes	yes	yes	yes

^a^ Strains designated with the same letter contain identical *hsdRMS* loci (see text and Fig 2).

^b^ The *hdsS* genes in SHL004 contain frameshift mutations [the central repeats are exchanged with poly(A) or poly(T) tracts] and are predicted to be non-functional.

^c^ ND, not determined, as the *sdrE* gene in SHL004 contains frameshift mutations (located within the second encoded *Cna_B* domain) and is predicted to be non-functional.

^d^ ND, not determined, due to gaps in the assembly in the repeat domain region.

### The sequenced ST8 strains were closely related to *L*. *monocytogenes* R479a

**Fig 2:**
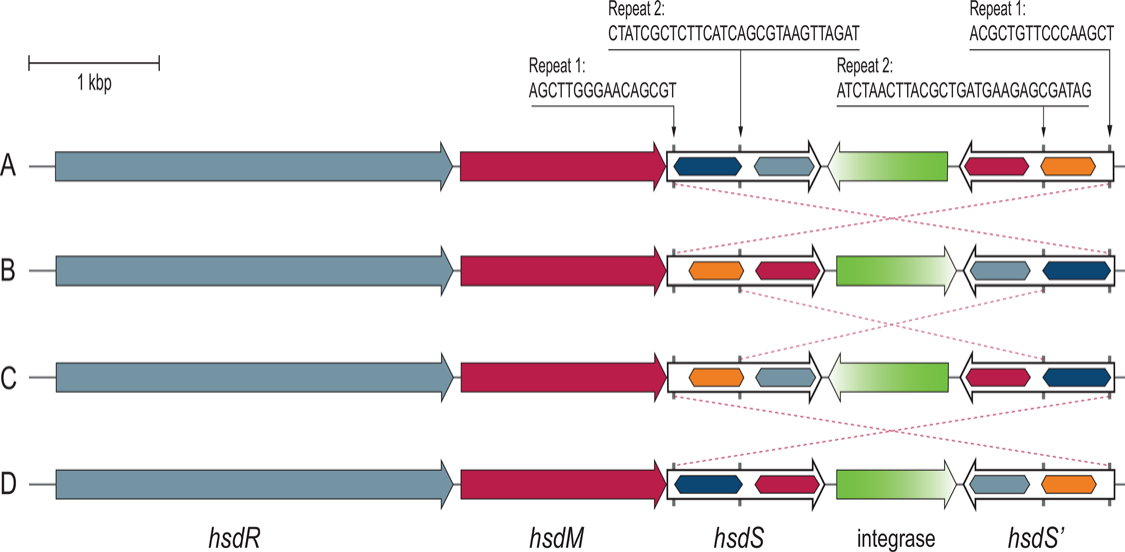
Invertible regions are present in the *hsd* locus encoding a Type I restriction modification system found in *L*. *monocytogenes* CC8 strains. The figure shows the four different configurations of the *hsd* locus, denoted variants A through D as indicated on the left, which were generated through site-specific gene shuffling. Within each DNA sequence recognition (specificity) gene (*hsdS*), the segments encoding each target recognition domain are shown as hexagonal boxes, in which identical colouring represents identical domains. The inverted repeat sequences are shown at the top, with arrows pointing to their location within the *hsdS* genes. Dotted lines drawn between two *hsd* locus variants illustrate the DNA inversion event required for transition between the two configurations. The locus corresponds to genes LMR479A_0528 to LMR479A_0532 in strain R479a, which are inserted between genes corresponding to *lmo0520*-*lmo0521* in *L*. *monocytogenes* EGD-e.

Comparative sequence alignment revealed a high level of overall genome conservation, not only for strains for which epidemiological data suggested close ancestral relationships (MF4245/MF3949/MF4077 and MF5369/MF5377), but between all six genomes. Actually, all six strains contained the same two prophages, at the same insertion sites, in addition to the pLMR479a plasmid. The six genomes were completely co-linear, except from one locus in which site-specific inversion (3.3 Kbp) appeared to have occurred within an *hsdRMS* restriction-modification (R-M) locus (discussed below; Fig 2, Table 3). The only other differences observed between strains were the copy numbers of various tandemly repeated units (Table 3), deletion of one gene on the pLMR479a plasmid in strain MF5369, and a total of 61 SNPs and indels (Fig 1, S1 and S2 Tables). The total difference across all six genomes was one SNP/indel every 49.3 Kb, i.e. 0.002% divergence.

### MF4245, MF3949, and MF4077 from the salmon processing facility represent a nearly unchanged resident strain

Comparative analysis showed that the three strains MF4245, MF3949, and MF4077, collected within a period of ten years in the salmon processing facility, were differentiated by 12 SNPs and one 1 bp indel (Fig 1, S1 and S2 Tables). Of these, seven mutations (five in MF3949 and two in MF4077) resulted in non-synonymous amino acid substitutions. The three strains also carried three different variants of the *hsdRMS* R-M locus (discussed below; Fig 2, Table 3). Apart from these differences, the genome sequences were identical, even the prophage and plasmid regions. When the detected single nucleotide mutations were compared with the R479a genome, one synonymous SNP was unique to the strain from 2001, while strains MF3949 and MF4077 obtained ten years later had ten and two unique variant positions, respectively (Fig 1, S1 and S2 Tables).

Eight identified SNPs collectively differentiate the three strains from the Norwegian salmon processing plant from the Danish R479a strain (Fig 1, S1 and S2 Tables). Thus nine SNPs differentiated R479a and MF4245, isolated five years apart. R479a was also differentiated from the three Norwegian strains in having a different variant of the R-M locus (discussed below; Fig 2, Table 3), and by having different copy numbers of 1) the non-coding (nc) RNA LhrC, 2) a 213 bp repeat in an intergenic region of the tRNA-Arg^TCT^ prophage, and 3) repeated C-term_anchor domains in internalin J (Table 3).

### Transfer of *L*. *monocytogenes* between poultry processing facilities *via* contaminated processing equipment

The two *L*. *monocytogenes* ST8 strains MF5369 and MF5377 were sampled at two different Norwegian poultry processing plants, in which contamination of one facility was suspected to be *via* a conveyor belt line relocated from the other facility. Comparison of the two genomes resulted in detection of two qualified SNPs differentiating the two strains (S1 and S2 Tables). The allelic state for both SNPs was shared among MF5369, R479a, and the three currently sequenced strains from the Norwegian salmon processing plant. The two SNPs were thus unique to MF5377, isolated in 2013 from the conveyor belt equipment after its relocation to the second poultry processing plant. Additional detected differences were a short (5–6 bp) polymorphism detected in an intergenic region of the *comK* prophage in MF5377 and loss of one repeated protein domain each from cell surface proteins SdrE and internalin J of MF5377 (Table 3). The only genetic difference unique to MF5369, isolated in 2011 at the facility from which the equipment originated, was the deletion of one gene in the plasmid (the gene corresponding to LMR479A_p0083). The two strains also carried two different variants of the *hsdRMS* R-M locus (discussed below; Fig 2, Table 3).

The sequenced strains from the two poultry processing facilities were more distantly related from strain R479a than the sequenced strains from the salmon processing facility (MF4245, MF3949, and MF4077). MF5369 and MF5377 shared a total of 38 SNPs differentiating them from the predicted last common ancestor of R479a, MF3949, MF4245, and MF4077 (Fig 1, S1 and S2 Tables). Of these, two SNPs were located in the plasmid homologous to pLMR479a.

### Comparative analysis with other ST8 genomes

The comparative genomic analysis of ST8 strains was extended to include four publicly available ST8 draft genome sequences, in addition to the Danish strain R479a and the five currently sequenced strains. These were *L*. *monocytogenes* SHL004, a clinical strain isolated from a hospitalized patient in Shanghai, China in 2008 [25], *L*. *monocytogenes* strains Lm_1823 and Lm_1889 isolated from Italian ricotta cheese in 2012 [20], and *L*. *monocytogenes* strain Lm21045 (Table 1). No information regarding persistence was available for these strains.

Variant calling analysis detected 11 SNPs and four 1 bp indels (four in adenine or thymine homopolymeric tracts) unique to the SHL004 genome, while eight SNPs, of which two were non-synonymous substitutions, were unique to Lm21045 (Fig 1, S1 and S2 Tables). Both SHL004 and Lm21045 contained the pLMR479a plasmid and the same prophage in the *comK* locus as R479a and the five currently sequenced strains. SHL004 also contained the tRNA-Arg^TCT^ prophage, which was missing from strain Lm21045.

When the genomes from the two strains Lm_1823 and Lm_1889 from Italian ricotta cheese were compared, four genetic differences were found in the chromosomal backbone sequences (Table 3, S2 Table). One of these was an insertion in Lm_1823 in one of the five variable number tandem repeat (VNTR) loci used for MLVA profiling (LMV7; S2 Table) [27], accounting for the different MLVA profiles of these two strains [20], and exemplifying the difficulties with using conventional subtyping methods to investigate relationships between different strains. Comparative sequence analysis furthermore detected 69 SNPs and two 1 bp indels differentiating the chromosomal backbone of the two Italian strains from the eight other examined ST8 genomes (Fig 1, S1 and S2 Tables).

### Different prophages and plasmids can be present in *L*. *monocytogenes* ST8 strains

**Fig 3:**
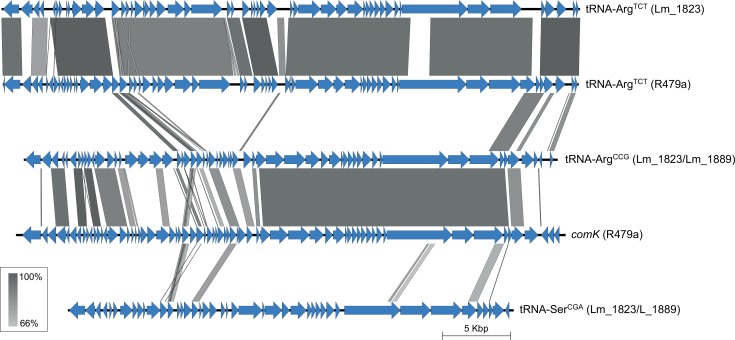
Comparison of prophages present in the *L*. *monocytogenes* ST8 genomes. Predicted ORFs are represented by blue arrows. Highly conserved segments, determined by pairwise BLASTn comparisons, are indicated by grey shaded regions with the color intensity indicating the nucleotide identity levels (from 66% to 100%). Similarities with E values lower than 0.001 were plotted. The prophages are denoted by names indicating the locus where they are inserted into the genome, followed by the host strain name(s) in parenthesis. For the prophages denoted by R479a, almost identical prophages were also identified in isolates MF4245, MF3949, MF4077, MF5369, MF5377 and SHL004, and in the case of the *comK* prophage, also in strain Lm21045.

While the chromosomal backbone sequences of the two Italian strains Lm_1823 and Lm_1889, isolated from cheese, were relatively similar to those of the eight other analysed ST8 genomes, the prophage and plasmid content was divergent in these two groups of strains. First of all, neither Lm_1823 nor Lm_1889 contained a prophage inserted into *comK*. Secondly, only Lm_1823 contained a prophage inserted downstream of the tRNA-Arg^TCT^ locus, and notably, this prophage was not identical to the tRNA-Arg^TCT^ prophage found in R479a, SHL004, and the Norwegian strains sequenced in the current study. Sequence coverage was only 88% with 92% nucleotide identity when the two phage sequences were aligned (Fig 3). Next, the genomes of Lm_1823 and Lm_1889 both contained prophages downstream of the genes encoding tRNA-Arg^CCG^ and tRNA-Ser^CGA^. Interestingly, a large part of the tRNA-Arg^CCG^ prophage was highly similar to the *comK* prophage present in the eight other analysed ST8 strains (91% nucleotide identity over ~65% sequence coverage; Fig 3). The tRNA-Ser^CGA^ prophage showed similarity to prophages present at the tRNA-Ser^CGA^ locus in other *L*. *monocytogenes* strains, e.g. those of the ST121 (CC121) strains 4423, 3253, and Lm_1880 (~97% identity; ~85% coverage), and the ST292 (CC8) strain 08–5578 (98.5% identity; ~85% coverage).

**Fig 4:**
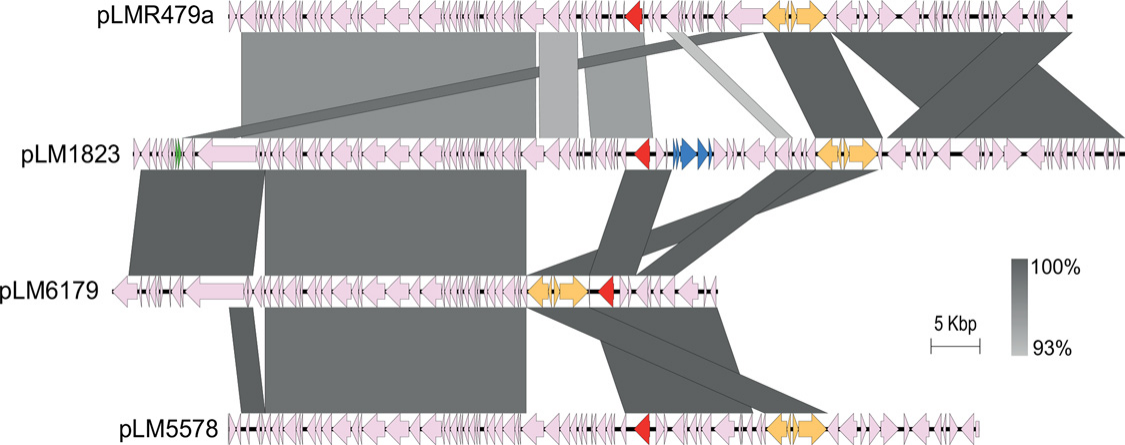
Comparison of *L*. *monocytogenes* plasmids. Predicted ORFs are represented by arrows, with the *repA* replication initiation gene in red, the *cadAC* cadmium resistance transposon Tn*5422* in orange, the *pemI*-*pemK* toxin-antitoxin module in green, and a predicted arsenic resistance operon in blue. Highly conserved segments, determined by pairwise BLASTn comparisons, are indicated by grey shaded regions with the color intensity indicating the nucleotide identity levels (from 93% to 100%). Similarities with E values lower than 0.001 and a minimum length of 1000 bp were plotted.

Finally, both Lm_1823 and Lm_1889 contained a plasmid of size ~101 Kbp, which was identical in the two strains except for one apparent rearrangement (for homologues of LMR479A_p0067-p0068) and one ~800 bp gap in the Lm_1889 genome assembly. The pLM1823 and pLM1889 plasmids were 97.5% identical to pLMR479a, in alignments covering 79% of pLM1823/pLM1889 and 92% of pLMR479a (Fig 4). Interestingly, large sections of the pLM1823 and pLM1889 plasmids showed higher levels of nucleotide identity towards the plasmids of the ST121 MLST subgroup than to the plasmids of the other analysed ST8 genomes, with over 99.3% identity towards the 62.2 Kbp plasmids of *L*. *monocytogenes* strains 6179 and Lm_1880 (pLM6179/pLM1880) in alignments covering 54% of pLM1823/pLM1889 and 87% of pLM6179/pLM1880 (Fig 4). They were also highly similar to the 77 Kbp plasmid pLM5579 from the ST292 (CC8) strain *L*. *monocytogenes* 08–5578, with over 99.9% identity in alignments covering 69% of pLM1823/pLM1889 and 91% of pLM5578. Among the unique genes of pLM1823/pLM1889, not present on the pLMR479a plasmid, were a *pemI*-*pemK* toxin-antitoxin stable maintenance module (X844_0080–81) [49, 50] and a locus which is predicted to confer resistance to arsenite (X844_0022–0026).

### Identification of a recombinogenic restriction-modification system

Comparison of the ST8 *L*. *monocytogenes* genomes revealed the presence of a Type I R-M locus (LMR479A_0528–0532) in which site-specific domain shuffling between DNA sequence recognition (specificity) genes (*hsdS*) had occurred. The locus was composed of one restriction gene (*hsdR*), one modification gene (*hsdM*), and two oppositely oriented *hsdS* genes flanking a gene encoding an integrase (Fig 2). Four different variants of the locus were identified within the examined ST8 genomes, which for clarity were denoted variants A, B, C, and D (see Table 3 and Fig 1). Sequence analysis of these variants indicated that inversions had occurred between two distinct recombination sequences located in the variable regions in the 5’-end and in the central part of the *hsdS* genes. These regions separate the two conserved domains of *hsdS* encoding the two domains of the specificity subunit which in Type I R-M systems each recognize one part of a bipartite target recognition sequence [51].

When the entire locus is considered, the recombination sequences form two pairs of inverted repeats (Fig 2). DNA inversion at the first repeat sequence, agcttgggaacagcgt, located in the 5’-end of *hsdS* results in inversion of a 3.3 Kbp element and switching between variants A and B or between variants C and D. DNA inversions of a 2.3 Kbp element between the second pair of inverted repeats, bearing the sequence ctatcgctcttcatcagcgtaagttagat, would result in switching between variants B and C or between variants A and D.

*L*. *monocytogenes* SHL004 contained a *hsd* locus with a variant D configuration, however in this strain, the locus contained *hsdS* genes with a 9 nt long poly(A) tract and a 10 nt long poly(T) tract, respectively, in place of the 29 nt long recombination sequence found in the central region of both *hsdS* genes in the eight other available ST8 genomes. Both mutations cause translational frameshifts resulting in insertion of TAA stop codons shortly downstream of the inserted homopolymer tract, causing premature termination in the middle of the putative SHL004 HsdS proteins. A mutation was also found in the *hsd* system in strain Lm_1889, which carried a short 5 bp polymorphism in the *hsdR* gene, resulting in premature termination likely to render the restriction endonuclease of this Type I R-M system non-functional (S2 Table). The analysed *L*. *monocytogenes* CC8 genomes of ST16, ST120, and ST292 (see Table 1) all contained *hsd* loci with intact variant D configurations, while the ST551 (CC8) *L*. *monocytogenes* strain Lm60 contained variant C (Fig 1).

The R-M system appears to be a phase variable locus which utilizes site-specific recombination to switch between four different DNA recognition site specificities. The integrase encoded between the two *hsdS* genes contains the active site residues of tyrosine recombinases, and presumably functions to generate the observed *hsdS* gene inversions. The observation that the identity of the allelic variant of the *hsd* locus was the only genetic trait that was discordant with the phylogeny of the ST8 strains defined by the genome-wide SNP analysis (Fig 1) indicates that site-specific domain shuffling actively occurs in the genomes of the ST8 strains.

### ST8 strains contain a RliB-CRISPR locus

A class of noncoding RNAs named CRISPRs (clustered regularly interspaced short palindromic repeats) has been shown to mediate resistance towards incoming bacteriophages and plasmids, and are usually processed by CRISPR-associated (*cas*) genes. A RliB-CRISPR locus which was processed not by Cas proteins but by the PNPase PnpA was previously identified in all sequenced *L*. *monocytogenes* strains [52]. The RliB-CRISPR loci found in the ten analysed ST8 strains were all identical with ten repeat sequences and nine spacers between homologues of genes LMR479A_0516 and LMR479A_0517. Identical *pnpA* sequences (LMR479A_1417) were also found in all ST8 genomes. No *cas* genes or additional CRISPR loci were identified in any of the examined ST8 genomes.

### Accessory genes encode R-M systems and bacteriophage resistance determinants

*L*. *monocytogenes* genomes are highly conserved and syntenic, with most accessory genes restricted to plasmids, prophages and so-called hypervariable hotspots [50]. Persistent strains may possibly share genetic features that are not present in non-persistent strains. To evaluate the accessory genome of the ST8 strains, outside of the prophage and plasmid regions, R479a was compared with the genomes of the commonly used lineage II laboratory strains *L*. *monocytogenes* EGD-e and 10403S [36]. The analysis showed that the unique sections of the ST8 chromosomes, relative to EGD-e and 10403S, contained 50 ORFs, distributed between ten genomic loci (S3 Table). Included in the set of accessory genes were two R-M systems. The first was the phase variable Type I R-M system (LMR479A_0528–0532) located between homologues of genes *lmo0520* and *lmo0521* in EGD-e (Fig 2). The second (LMR479A_1125–1126) was a Type III R-M system inserted into one of the described hypervariable hotspots [50], downstream of the homologue of gene *lmo1096* in EGD-e. The latter locus also encoded a protein (LMR479A_1133) with predicted function in abortive resistance to bacteriophage infection [53]. A third locus also predicted to encode bacteriophage resistance by abortive infection (LMR479A_2137–2138) was located in another hypervariable hotspot, between homologues of genes *lmo2025*-*lmo2028* in EGD-e. The remaining accessory genes for which a function could be predicted comprised genes encoding a putative ATP-binding cassette protein (LMR479A_0030), one locus encoding proteins predicted to be involved in carbohydrate transport (LMR479A_0269–0274), and one metallopeptidase (LMR479A_2720) (S3 Table).

### Virulence and resistance genes in ST8 genomes

Of the 78 putative virulence-associated genes previously reported in *L*. *monocytogenes* EGD-e [54], all but four (homologues to *lmo0320*, *lmo1099*, *lmo1102*, and *lmo2026*) were present in the ST8 strains. Of note, all examined strains encode full-length internalin A proteins, which is a principal virulence factor important for host cell invasion and colonization [55]. Lm_1823/Lm_1889 harbor *inlA* allele 2 as defined by Ragon *et al*. [24], while the remaining seven analysed ST8 strains harbor *inlA* allele 1 (these two alleles differ by one SNP).

The extrachromosomal plasmids of the ST8 strains carry several genes putatively involved in heavy metal resistance, including genes putatively involved in copper detoxification (LMR479A_p0067-p0068) [56] and the Tn*5422* transposon carrying a cadmium efflux system (LMR479A_p0062-p0065) [57]. However, the ST8 genomes do not contain the *bcrABC* resistance cassette or the Tn*6188* transposon harboring the *qacH* gene, which are known to confer tolerance to the disinfectant benzalkonium chloride [58, 59].

## Discussion

### Evolutionary relationships between ST8 strains revealed using genome-wide SNP analysis

Comparative genome analysis at the SNP level was used in the current study to infer evolutionary relationships between a set of *L*. *monocytogenes* strains belonging to the MLST ST8 subtype. Four of the analysed strains; R479a, MF4245, MF3949, and MF4077, were sampled in two different salmon processing plants in Denmark and Norway over a time-span of 15 years, and differed by 61 SNPs and indels. Two strains; MF5369 and MF5377, were isolated two years apart in two different poultry processing facilities in Norway, and differed by two qualified SNPs. The observed divergence in each group was comparable to that observed in several studies investigating *L*. *monocytogenes* outbreaks: In the study by Orsi *et al*. [9], four *L*. *monocytogenes* strains derived from a clone persisting in a food processing facility over 12 years differed by 11 SNPs outside of the *comK* prophage region, while Gilmour *et al*. [8] showed that 28 SNPs differentiated two clinical isolates from an outbreak of listeriosis. In a study by Stasiewicz *et al* [14], in which 188 isolates from a longitudinal study of *L*. *monocytogenes* in retail delis were studied by WGS, median SNP counts within isolates of putative persistent strains ranged from 1 to 13 SNPs. The level of divergence observed within each of the two groups of strains examined in the current study is thus consistent with each group having originated from a relatively recent common ancestor.

*L*. *monocytogenes* MF4245, isolated in 2001 at the Norwegian salmon processing facility, contained one SNP not found in MF3949 or MF4077 isolated a decade later. MF3949 and MF4077 were therefore not likely to be direct descendants of MF4245. Similarly, *L*. *monocytogenes* MF5369 isolated in a poultry processing plant in 2011 lacked one plasmid gene which was present in all other analysed ST8 genomes, indicating that MF5369 was not a direct ancestor of MF5377, isolated in 2013. However, the lack of detection of a direct line of descent within each group of isolates does not imply that they do not represent resident strains harbored in or transferred between the food processing facilities in question. Firstly, the unique mutations in MF4245 and MF5369 could have been acquired during storage, sampling, isolation or subculture in the laboratory. Also, a resident strain established in a given food processing environment is likely to represent a source population of bacteria harboring some extent of genetic diversity [60]. Potentially, the single recent ancestor of each of the two groups of strains sequenced in the current study could have been identified if a larger set of isolates from the food processing facilities had been subjected to WGS. This illustrates the point that the full advantage of using WGS and SNP analysis for tracking routes of pathogen transmission is only realized when genomic data from a sufficient sample of the diversity is available [7, 60, 61].

### Transmission of *L*. *monocytogenes* ST8 between food processing environments

R479a was identified as persisting in the Danish salmon processing plant from 1996 to 1999 based on recurrent isolation of *L*. *monocytogenes* with the same RAPD profile [15, 16]. The close genetic identity between R479a and the three sequenced *L*. *monocytogenes* strains from the Norwegian salmon processing facility, in addition to repeated detection after cleaning and sanitation of isolates of the same MLVA profile as the sequenced strains, suggests that the persistent strain found in the Danish facility also has persisted in the Norwegian salmon processing facility over a period of at least 13 years. It is not known whether there was any contact between the Norwegian and Danish facilities in the years preceding the first detection of this clone in either facility, which could indicate a direct transmission of the contamination between the two locations. Notably, however, raw materials at both facilities include slaughtered ocean-farm raised salmon from Norway [15]. During sampling in the Danish facility, *L*. *monocytogenes* with the same RAPD profile as R479a was on one occasion detected in a sample taken from raw salmon [15], while in the Norwegian facility, isolates with the same MLVA profile as MF4245, MF3949, and MF4077 were detected on raw salmon from two suppliers. Hypothetically, there may exist a third facility (for example a Norwegian salmon slaughterhouse) also harboring the persistent strain, constituting the source of the contamination in both the Norwegian and Danish processing plants. Typing of *L*. *monocytogenes* from processed raw materials should be recommended to reveal if processing plants earlier in the production chain harbour persistent strains.

The sequenced strains from salmon and poultry processing environments were sufficiently closely related (37 SNPs differentiated the two groups; Fig 1, S1 and S2 Tables) to be expected to have similar phenotypes, including an ability to become established in food processing environments. In at least one previous case, *L*. *monocytogenes* contamination with a particular PFGE subtype was suggested to have been transferred between food processing sites as a result of relocation of processing equipment [62]. A divergence of two SNPs between the strains recovered two years apart from the two poultry processing facilities is consistent with the hypothesis of a direct transmission of contamination between the two facilities. Further analysis of *L*. *monocytogenes* isolates sampled from the plant in which the conveyor belt line originated would be required to determine whether MF5369 is representative of a resident *L*. *monocytogenes* contamination in that plant. However, it should be stressed that transferring used equipment between production plants represents a risk for introduction of *L*. *monocytogenes*.

The current study provides two case examples where almost identical *L*. *monocytogenes* strains were detected in more than one food processing facility. Globalisation and a demand for increasingly efficient food production and processing result in progressively complex food chains, increasing the risk of transmission of single clones of pathogens to multiple processing facilities. In this scenario, epidemiological data linking infections back to specific food samples, rather than to processing environments, seems crucial for accurate interpretation of data from WGS-based typing of bacterial isolates during outbreak investigations. Increased public availability of genome data obtained from systematic pathogen surveillance in food industry environments would undoubtedly increase the accuracy of transmission tracking during an outbreak scenario [6, 7, 61].

### Conservation of the accessory genome

Phylogenetically, the ten *L*. *monocytogenes* ST8 genomes analysed in the current study separated into two clades (Fig 1). Within each clade, prophage and plasmid sequences were conserved at the same level as the core genome. A high degree of conservation of prophage and plasmid sequences was also noted by Schmitz-Esser *et al*. [13] for prophage sequences found within the genomes of the persistent *L*. *monocytogenes* ST121 subtype. As prophage diversification is considered to represent the major driver of short term evolution of *L*. *monocytogenes* [8, 9, 63], the observed level of conservation in mobile genetic regions seems remarkably high. However, the content of prophage and plasmid sequences differed significantly between the two clades of ST8 strains. Diversity in prophage content between closely related *L*. *monocytogenes* strains was also observed in several previous studies [8, 9, 50]. Further analysis in which a wider diversity of *L*. *monocytogenes* genomes is included would be required to firmly establish whether or not the conservation of mobile genetic elements is significantly enhanced in the ST8 and ST121 subgroups of *L*. *monocytogenes* compared with in other sets of closely related strains.

It was proposed by Schmitz-Esser *et al*. [13] that the high degree of similarity between prophages and plasmids in different *L*. *monocytogenes* ST121 strains derived from both fish, meat, and cheese production facilities may be a result of these strains having adapted to niches common to food processing environments, suggesting that selective pressure had resulted in conservation of prophages and plasmids between strains. A somewhat contrasting notion was put forth by Verghese *et al*. [63], suggesting that diversification within genes in prophage regions would allow *L*. *monocytogenes* strains to rapidly adapt to niches specific to different food processing facilities and foods. The current study provides one example for each side of the argument, as persistent ST8 strains from salmon and poultry processing environments were basically genetically identical, while their content of prophage and plasmid sequences was significantly divergent from that found in the genomes of the two ST8 strains associated with a cheese production facility. Possibly, genetic factors may render certain strains of *L*. *monocytogenes* more prone to bacteriophage predation and horizontal gene transfer, resulting in differences between subtypes in the rate of exchange of the accessory genome. Bacterial resistance mechanisms against foreign DNA and bacteriophage infection include decreased availability of phage receptors, the presence of R-M systems that can destroy invading DNA and abortive infection (Abi) systems that lead to death of the infected cell [64, 65]. The accessory genomes of the currently examined ST8 strains harbour two R-M systems and three genes with predicted Abi functions (S3 Table) which could render the ST8 group of genomes resilient to incoming mobile genetic elements. Of note, one of the R-M systems, encoded by a *hsdRMS* locus, was predicted to show phase variable recognition sequence specificity (Fig 2). Similar systems have not previously been described in *L*. *monocytogenes*, but have been found in other species, including in *Bacteroides fragilis* and *Mycoplasma pulmonis* [66, 67]. The ST8 strains also harbor a RliB-CRISPR system likely to protect against bacteriophage infection [52]. However, only one of the strains, MF3949, harbors a mutation which is predicted to mediate phage resistance through adsorption inhibition. This strain has a nonsense mutation in *csbB* (LMR479A_2678/lmo2550) encoding a glycosyl transferase shown to be necessary for decoration of wall teichoic acid with N-acetylglucosamine residues [68], and for which disruptive mutations was recently shown to mediate resistance to certain virulent phages [69].

As an alternative hypothesis, observed differences in the rates of divergence of the accessory genome in different populations of *L*. *monocytogenes* could be accounted for, at least in part, by environmental factors rather than genetic factors. For a given *L*. *monocytogenes* strain, the rate of exchange of mobile genetic elements, such as plasmids and prophage sequences, would be expected to be dependent on the availability of compatible sequences in the environment in which the strain resides. The second group of ST8 strains analysed in the current study, Lm_1823 and Lm_1889, were sequenced as part of an epidemiological investigation of a listeriosis outbreak associated with an Italian cheese product, in which 430 *L*. *monocytogenes* isolates were genotyped and found to belong to six different MLVA profiles [20]. It is interesting to note that the Lm_1823 and Lm_1889 plasmids show higher nucleotide identity towards the plasmids of the *L*. *monocytogenes* ST121 subgroup than towards the pLMR479a-type plasmids (Fig 4), especially since one of the strains sequenced as part of the cheese-related outbreak investigation was the *L*. *monocytogenes* ST121 strain Lm_1880 [13, 20]. Furthermore, of the analysed ST8 genomes, only Lm_1823 and Lm_1889 contain a prophage inserted into the tRNA-Ser^CGA^ locus, and this prophage was highly similar to the one found at the same locus in the Lm_1880 genome. It is thus tempting to speculate that exchange of plasmid and prophage sequences may have occurred in the Italian cheese-related *L*. *monocytogenes* genomes as a result of a high level of access to compatible mobile genetic elements, including a high phage pressure, in the environment in which they recently evolved.

### ST8 strains have high pathogenic potential

It has been suggested that on average, persistent strains may be less virulent than sporadic strains [2, 70]. Strains of the persistent ST121 subtype encode truncated internalin A (InlA) proteins [13], a feature which is associated with attenuated virulence and which is often found in *L*. *monocytogenes* isolates from food processing environments [71, 72]. In contrast, ST8 strains harbor full-length *inlA* genes, and all but four of 78 putative virulence-associated genes previously reported for *L*. *monocytogenes* EGD-e [54]. ST8 strains are commonly reported to be responsible for cases of human listeriosis. For example, a human clinical strain, SHL004, was among the nine ST8 genomes analysed in the current study [25], and ST8 was the most prevalent MLST subtype found among human strains isolated from patients with severe illness in Switzerland in 2011–2013 [26]. In contrast, when *L*. *monocytogenes* strains from food samples isolated in Switzerland in 2011–2014 were studied, ST9 and ST121 were the most frequently detected STs [21]. ST8 strains are also closely related to the ST120 subtype (both belong to CC8) which has been implicated in several cases of foodborne outbreaks [8, 22]. ST8 strains are therefore predicted to have high pathogenic potential. Their persistence in food processing facilities represents a serious threat to food safety, as food products are the primary source for human listeriosis infection, and contamination of foods is typically from sources in the processing plant environment [4, 73].

## Conclusions

The present study demonstrates that WGS and SNP-based analysis is well-suited to answer questions regarding persistence and epidemiology of *L*. *monocytogenes* in food processing facilities and in the food chain. Our results suggest that ST8 is a widespread conserved subtype of *L*. *monocytogenes* with a high pathogenic potential and an ability to become resident in different food production environments.

## Supporting Information

S1 Table Summary of SNPs and indels showing the distribution of nucleotide states in all analysed CC8 *L*. *monocytogenes* strains. (XLSX)

S2 Table SNPs and indels detected in *L*. *monocytogenes* ST8 isolates. The allelic state for each SNP/indel is presented relative to that found in the neighbour *L*. *monocytogenes* strains 10304S, 08–5578, and 08–5923. Plasmid and prophage regions in *L*. *monocytogenes* isolates Lm_1823 and Lm_1889 are disregarded.
(PDF)

S3 Table Accessory genome in ST8 isolates relative to *L*. *monocytogenes* strains EGD-e and 10403S. Genes in prophages and plasmids are disregarded.
(PDF)
