# Supplementary material for: Genome Analysis of Listeria monocytogenes Sequence Type 8 Strains Persisting in Salmon and Poultry Processing Environments and Comparison with Related Strains
Source: PLoS One. 2016 Mar 8;11(3):e0151117. doi: 10.1371/journal.pone.0151117 (PMC4783014; doi:10.1371/journal.pone.0151117)
Supplement: S2 Table — The allelic state for each SNP/indel is presented relative to that found in the neighbour L. monocytogenes strains 10304S, 08–5578, and 08–5923. Plasmid and prophage regions in L. monocytogenes isolates Lm_1823 and Lm_1889 are disregarded. (PDF) [file pone.0151117.s002.pdf]

S2 Table (page 1 of 4): SNPs and indels detected in *L. monocytogenes* ST8 isolates. The allelic state for each SNP/indel is presented relative to that found in the neighbour *L. monocytogenes* strains 10304S, 08-5578, and 08-5923. Plasmid and prophage regions in *L. monocytogenes* isolates Lm_1823 and Lm_1889 are disregarded.

	SNP Position (R479a)	Locus tag (R479a)	Protein length (amino acids)	Protein function (listed for non-synonymous substitutions)	Nucleotide change	Amino acid change
Variants common to R479a, MF4245, MF3949, MF4077, SHL004, Lm21045, MF5369 and MF5377						
	16 488	LMR479A_0013	368 aa	Cytochrome aa3-600 quinol oxidase (subunit II)	T→G	^{90}Phe→Leu
	294 717	LMR479A_0287	211 aa	Histidine phosphatase superfamily protein	G→A	^{87}Met→Ile
	445 338	LMR479A_0443	800 aa	Internalin A	C→T	^{192}Ser→Phe
	593 818	LMR479A_0588	487 aa	Conserved protein of unknown function, with Cell-wall surface anchor repeat domains	G→A	^{154}Val→Ile
	760 445	LMR479A_0773	203 aa	Conserved protein of unknown function	C→T	^{52}Ser→Phe
	768 185	LMR479A_0781	418 aa	Putative GTP-binding protein, GTPase HflX family protein	G→T	^{318}Ala→Ser
	897 886	LMR479A_0900	189 aa	Putative exported adhesion protein (homologue to EGD-e *lmo0880*)	TT→T	Frameshift mutation at codon 168 resulting in premature termination at codon 190
	1 346 148	LMR479A_1395	434 aa	Conserved protein of unknown function DUF3440	A→T	^{108}Trp→Arg
	1 384 317	LMR479A_1437	488 aa	Glycine decarboxylase (subunit 2)	C→A	^{46}Pro→Gln
	1 782 613	LMR479A_1815	410 aa	Aminopeptidase	G→C	^{80}Ala→Pro
	2 802 215	LMR479A_2861	147 aa	Conserved protein with methyltransferase domain	A→T	^{136}Ser→Thr
	2 880 025	LMR479A_2930	99 aa	Conserved exported protein of unknown function	G→C	^{26}Ala→Gly
	24 172	LMR479A_0019 ↔ LMR479A_0020			G→C	noncoding
	692 692	LMR479A_0696			G→A	synonymous
	786 365	LMR479A_0801			C→T	synonymous
	840 061	LMR479A_0848← LMR479A_0849			T_9→T_8	noncoding
	845 621	LMR479A_0855			C→T	synonymous
	1 045 439	LMR479A_1063			G→A	synonymous
	1 057 028	LMR479A_1079→ LMR479A_1080			C→T	noncoding
	1 406 210	LMR479A_1460			G→A	synonymous
	1 663 673	LMR479A_1705			G→T	synonymous
	2 429 990	LMR479A_2477			T→C	synonymous
	2 687 274	LMR479A_2731→← LMR479A_2732			G→A	noncoding
Variants common to R479a, MF4245, MF3949, MF4077, SHL004, and Lm21045						
	122 794	LMR479A_0119	235 aa	EAL domain-containing phosphodiesterase PdeD	G→A	^{221}Val→Ile
	2 873 741	LMR479A_2923	327 aa	ADP-ribosylation/Crystallin J1 family protein	C→G	^{24}Cys→Ser
Variants common to R479a, MF4245, MF3949 and MF4077						
	2 096 262	LMR479A_2124	885 aa	Putative alpha-mannosidase MngB	G→A	^{795}Ala→Val
	2 220 337	LMR479A_2241	607 aa	Putative metabolite transporter	G→T	^{208}His→Asn
	2 605 097	LMR479A_2651	331 aa	MreB-like cell shape determining protein	G→A	^{63}Pro→Leu
	126 081	LMR479A_0125			A→G	synonymous
	1 251 584	LMR479A_1290→ LMR479A_1291		Note: Located in tRNA-Arg^{TCT} prophage (not present in Lm21045, Lm_1823 and Lm_1889)	T→C	noncoding
	2 155 786	LMR479A_2180↔ LMR479A_2181			A→G	noncoding
	2 208 238	LMR479A_2232			A→G	synonymous
	2 520 292	LMR479A_2571↔ LMR479A_2572			T→A	noncoding
	2 822 750	LMR479A_2881↔ LMR479A_2882			T→A	noncoding
Variants unique to R479a						
	883 111	LMR479A_0884	557 aa	Phosphoglucomutase	T→G	^{304}Val→Gly
	1 388 589	LMR479A_1441	353 aa	Putative aminopeptidase	T→C	^{344}Ile→Thr
	2 128 656	LMR479A_2151	738 aa	Penicillin-binding protein 2B	C→G	^{323}Ala→Pro
	2 302 826	LMR479A_2320	866 aa	ATP-dependent protease ClpB	A→T	^{74}Ser→Thr
	368 857	LMR479A_0359			A→C	synonymous
	1 252 780	LMR479A_1293→ LMR479A_1294		Note: Located in tRNA-Arg^{TCT} prophage (not present in Lm21045, Lm_1823 and Lm_1889)	C→T	noncoding
	1 389 453	LMR479A_1443			T→C	synonymous
Variant common to MF4245, MF3949 and MF4077						
	326 353	LMR479A_0318			G→A	synonymous
Variant unique to MF4245						
	2 245 825	LMR479A_2268			C→T	synonymous

S2 Table, continued (page 2 of 4):

	SNP Position (R479a)	Locus tag (R479a)	Protein length (amino acids)	Protein function (listed for non-synonymous substitutions)	Nucleotide change	Amino acid change
Variants unique to MF3949						
	114 570	LMR479A_0113	756 aa	Putative chitinase	A_5→A_4	Frameshift mutation at codon 496 resulting in premature termination at codon 504
	388 086	LMR479A_0381	237 aa	Putative transcriptional regulator (GntR family HTH domain)	G→A	^{65}Ala→Val
	1 136 271	LMR479A_1157	382 aa	Predicted immunity protein 14 family protein	G→T	^{305}Glu→Asp
	2 080 048	LMR479A_2109	354 aa	Conserved protein with sugar isomerase domains	A→G	^{139}Leu→Ser
	2 627 502	LMR479A_2678	315 aa	Putative glycosyltransferase 2,CsbB	T→A	Nonsense mutation at codon ^{149}Lys
	15 888	LMR479A_0012→ LMR479A_0013			A→G	noncoding
	744 990	LMR479A_0753			G→A	synonymous
	1 322 132	LMR479A_1372→ LMR479A_1373			G→T	noncoding
	2 648 729	LMR479A_2696← LMR479A_2697			G→A	noncoding
	2 834 901	LMR479A_2892			G→A	synonymous
Variants unique to MF4077						
	1 643 093	LMR479A_1685	343 aa	N-acetylglutamate gamma-semialdehyde dehydrogenase, ArgC	C→T	Nonsense mutation at codon ^{219}Trp
	1 759 340	LMR479A_1786	313 aa	Conserved protein of unknown function (NAD-dependent 2-hydroxyacid dehydrogenase domain)	G→A	^{198}Pro→Leu
Variants unique to SHL004						
	74 820	LMR479A_0070	160 aa	Conserved protein of unknown function	A_9→A_8	Frameshift mutation at codon 13 resulting in premature termination at codon 28
	220 906	LMR479A_0223	1179 aa	Transcription-repair coupling factor	C→T	^{27}Ala→Val
	270 600	LMR479A_0272	276 aa	Maltose/maltodextrin transport system permease protein	T→G	^{64}Phe→Val
	283 803	LMR479A_0279	486 aa	Aryl-phospho-beta-d-glucosidase	C→T	^{278}Thr→Ile
	490 642	LMR479A_0490	159 aa	Conserved protein with GyrI-like small molecule binding domain	T_5→T_6	Frameshift mutation at codon 21 resulting in premature termination at codon 23
	1 375 444	LMR479A_1425	322 aa	Glucose kinase	G→A	^{90}Gly→Glu
	1 450 157	LMR479A_1499 (overlaps with LMR479A_1500)	49 aa	Protein of unknown function	A→G	^{3}Leu→Pro
		LMR479A_1500 (overlaps with LMR479A_1499)	75 aa	Putative transcriptional regulator (Cro/C1-type HTH domain)		^{41}Ser→Gly
	1 553 799	LMR479A_1604	246 aa	Conserved exported protein with alpha/beta hydrolase fold	G→A	^{5}Ala→Val
	2 124 420	LMR479A_2148	455 aa	UDP-N-acetylmuramoylalanyl-D-glutamate ligase	C→T	^{59}Val→Ile
	2 221 835	LMR479A_tRNA14		tRNA-Lys	C→T	mutation in tRNA
	2 261 712	LMR479A_2285	455 aa	Putative regulatory protein (sigma-54 factor interaction domain and Fis-type HTH domain)	GG→G	Frameshift mutation at codon 201 resulting in premature termination at codon 240
	2 728 883	LMR479A_2787	688 aa	Putative transcriptional antiterminator protein	C→T	^{25}Glu→Lys
	202 968	LMR479A_0204→ LMR479A_0205			T_8→T_7	noncoding
	1 462 667	LMR479A_1511			C→A	synonymous
	2 344 668	LMR479A_2364			T→C	synonymous
Variants unique to Lm21045						
	905 701	LMR479A_0910	278 aa	Anti-sigma B factor antagonist; component of the stressosome	C→T	^{77}Leu→Phe
	1 588 836	LMR479A_1637	266 aa	ATP binding protein MinD; activator of MinC	G→A	^{194}His→Tyr
	217 460	LMR479A_0218← LMR479A_0219			G→A	noncoding
	429 729	LMR479A_0427			A→G	synonymous
	743 515	LMR479A_0751→ LMR479A_0752			T→G	noncoding
	1 284 339	LMR479A_1331→ ← LMR479A_1332			G→A	noncoding
	1 538 406	LMR479A_1590			A→T	synonymous
	2 437 733	LMR479A_2484			C→T	synonymous

S2 Table, continued (page 3 of 4):

	SNP Position (R479a)	Locus tag (R479a)	Protein length (amino acids)	Protein function (listed for non-synonymous substitutions)	Nucleotide change	Amino acid change
Variants common to MF5369 and MF5377						
	81 788	LMR479A_0080	322 aa	Conserved protein of unknown function	A→T	^{318}Asn→Tyr
	89 881	LMR479A_0091	327 aa	Aldo/keto reductase family protein	G→C	^{306}Ala→Gly
	191 404	LMR479A_0193	553 aa	Trehalose-6-phosphate hydrolase	G→T	Nonsense mutation at codon ^{171}Glu
	336 167	LMR479A_0328	224 aa	Thiaminase II family protein	T→A	^{94}Leu→Gln
	586 074	LMR479A_0581	393 aa	ATP phosphoribosyltransferase regulatory subunit	C→T	^{224}Ala→Thr
	635 972	LMR479A_0629	583 aa	Glycerophosphoryl diester phosphodiesterase	C→A	^{501}Pro→Gln
	721 085	LMR479A_0725	429 aa	Flagellar capping protein FliD	C→A	^{188}Ala→Glu
	866 401	LMR479A_0867	616 aa	Amidase	G→A	Nonsense mutation at codon ^{490}Gln
	1 216 789	LMR479A_1247	1066 aa	Putative membrane transport protein	A→C	^{94}Thr→Pro
	1 575 765	LMR479A_1623	379 aa	tRNA-guanine transglycosylase	A→T	^{168}Ser→Ser
	1 641 346	LMR479A_1683	250 aa	Acetylglutamate kinase	C→T	Nonsense mutation at codon ^{50}Glu
	1 713 743	LMR479A_1752	164 aa	Putative cell surface metallopeptidase	T→C	^{25}Asp→Gly
	2 343 482	LMR479A_2362	89 aa	Predicted protein	G→T	^{32}Arg→Ile
	2 433 908	LMR479A_2480	927 aa	Transmembrane protein	G→C	^{210}Leu→Val
	2 812 935	LMR479A_2871	882 aa	Putative glycoside hydrolase	A→T	^{803}Asn→Ile
	2 820 402	LMR479A_2878	402 aa	Putative metabolite efflux transporter, major facilitator superfamily	A→C	Nonsense mutation at codon ^{80}Leu
	p22414	LMR479A_p0022	190 aa	Note: in plasmid (not present in Lm_1823 and Lm_1889). Conserved protein of unknown function	T→G	^{101}Lys→Thr
	p43471	LMR479A_p0049	165 aa	Conserved protein of unknown function. Note: in plasmid (not present in Lm_1823 and Lm_1889)	G→A	^{33}Arg→Cys
	188 598	LMR479A_0191→ LMR479A_0192			G→A	noncoding
	304 677	LMR479A_0297			C→T	synonymous
	807 840	LMR479A_0818			G→A	synonymous
	1 218 015	LMR479A_1247			C→T	synonymous
	1 605 295	LMR479A_1652			A→T	synonymous
	1 639 027	LMR479A_1681			C→A	synonymous
	1 836 744	LMR479A_1863			T→C	synonymous
	2 392 458	LMR479A_2420		Note: Located in *comK* prophage (not present in Lm_1823 and Lm_1889)	C→T	synonymous
	2 803 632	LMR479A_2862			A→G	synonymous
Variants unique to MF5377						
	111 554	LMR479A_0110	96 aa	Conserved protein with antibiotic biosynthesis monooxygenase-like domain	G→A	Nonsense mutation at codon ^{56}Trp
	1 870 855	LMR479A_1898	156 aa	Putative transcriptional regulator (MerR-type HTH domain)	T→G	^{57}His→Gln
Variants unique to Lm_1823						
	859 967	LMR479A_0860	2047 aa	Peptidoglycan binding protein. Note: One of the VNTR loci used for MLVA profiling (locus LMV7) is located in this gene (Lindstedt et al., 2008). The indicated mutation results in altered MLVA subtype.	C→CCAAAAT ATC	Increases the number of CCT-ATA-AAA (Pro-Ile-Lys) repeats from 5 to 6
	2 867 916	LMR479A_2920	431 aa	Phosphotransferase system, cellobiose-type IIC component	A→T	^{341}Val→Glu
Variant unique to Lm_1889						
	529 380	LMR479A_0528	1020 aa	Type I restriction endonuclease HsdR	ATTAG→ CTAAT	^{806}Gln→His and nonsense mutation at codon ^{807}Leu

S2 Table, continued (page 4 of 4):

	SNP Position (R479a)	Locus tag (R479a)	Protein length (amino acids)	Protein function (listed for non-synonymous substitutions)	Nucleotide change	Amino acid change
Variants common to Lm_1823 and Lm_1889						
	27 108	LMR479A_0024	275 aa	Phosphotransferase system (PTS), mannose/fructose/sorbose family IID component	G→A	^{157}Val→Ile
	78 406	LMR479A_0075	151 aa	Conserved protein of unknown function	G→T	Nonsense mutation at codon ^{109}Glu
	252 873	LMR479A_0248	234 aa	2-C-methyl-D-erythritol 4-phosphate cytidylyltransferase	G→T	^{161}Val→Phe
	268 746	LMR479A_0270	408 aa	Maltose/maltodextrin transport system substrate-binding protein	G→A	^{307}Ala→Thr
	330 768	LMR479A_0321	463 aa	6-phospho-beta-galactosidase	T→C	^{78}Lys→Arg
	357 236	LMR479A_0344	1778 aa	Internalin I	G→A	^{1140}Val→Ile
	424 925	LMR479A_0421	867 aa	putative PEP-dependent enzyme	G→A	^{18}Leu→Phe
	480 884	LMR479A_0473	58 aa	Conserved protein of unknown function	G→A	^{11}Gly→Glu
	682 129	LMR479A_0681	288 aa	HAD-superfamily hydrolase protein	C→T	^{246}Met→Ile
	822 736	LMR479A_0836	876 aa	Calcium-transporting ATPase	G→A	^{140}Gly→Glu
	860 558	LMR479A_0861	85 aa	Conserved protein of unknown function DUF1294	T→C	^{20}Ile→Val
	872 904	LMR479A_0875	470 aa	UDP-N-acetylmuramoylalanyl-D-glutamyl-2,6-diaminopimelate-D-alanyl-D-alanine ligase	G→A	^{248}Gly→Ser
	951 071	LMR479A_0957	169 aa	2'-O-methyltransferase TrmL	A→T	Mutation at stop codon resulting in fusion with downstream gene LMR479A_0958
	1 132 620	LMR479A_1153	571 aa	Uncharacterized ABC transporter ATP-binding protein	A→T	^{471}Gln→Leu
	1 315 314	LMR479A_1367	290 aa	Aldose 1-/Glucose-6-phosphate 1-epimerase family protein	T→G	^{151}Asp→Glu
	1 327 345	LMR479A_1375	622 aa	Putative peptidoglycan O-acetyltransferase	C→G	^{279}Met→Ile
	1 373 081	LMR479A_1422	179 aa	5-formyltetrahydrofolate cyclo-ligase family protein	C→T	^{111}Thr→Ile
	1 485 204	LMR479A_1531	235 aa	Putative branched-chain amino acid transport, permease; AzlC family	T→A	^{180}Leu→Ile
	1 729 063	LMR479A_1765	1711 aa	Peptidoglycan linked protein (LPXTG)	C→A	^{1155}Val→Phe
	1 840 812	LMR479A_1865	225 aa	(S)-3-O-geranylgeranylglyceryl phosphate synthase	C→T	^{122}Ala→Thr
	1 926 560	LMR479A_1952	274 aa	Conserved protein with STAS domain	G→A	^{230}Gly→Ser
	1 951 211	LMR479A_1979	327 aa	Conserved protein of unknown function DUF939	C→A	^{11}Ala→Ser
	2 001 328	LMR479A_2027	421 aa	Putative NAD(FAD) dehydrogenase	C→T	^{168}Val→Ile
	2 156 041	LMR479A_2181	228 aa	CAAX amino terminal protease family protein	T→C	^{84}Leu→Arg
	2 316 452	LMR479A_2335	907 aa	Conserved protein with P-loop NTPase domains	G→T	^{301}Asn→Lys
	2 366 003	LMR479A_2383	78 aa	Conserved protein with IDEAL domain	G→C	^{24}Thr→Arg
	2 415 961	LMR479A_2462	440 aa	Putative monooxygenase	G→A	Nonsense mutation at codon ^{337}Arg
	2 543 393	LMR479A_2592	477 aa	Exported putative carbohydrate-binding protein	C→T	^{18}Val→Ile
	2 565 167	LMR479A_2613	956 aa	UvrABC system subunit A	C→T	^{627}Ala→Thr
	2 662 606	LMR479A_2709	222 aa	Putative hemin import ATP-binding protein HrtA	C→T	^{20}Glu→Lys
	2 860 047	LMR479A_2912	279 aa	Transcription antiterminator LicT	A→C	^{80}Phe→Cys
	268 787	LMR479A_0270			G→A	synonymous
	442 643	LMR479A_0440↔ LMR479A_0441			C→T	noncoding
	546 025	LMR479A_0541			G→A	synonymous
	580 135	LMR479A_0572← LMR479A_0573			C→T	noncoding
	615 311	LMR479A_0606			G→A	synonymous
	735 733	LMR479A_0742			C→T	synonymous
	864 258	LMR479A_0865			C→T	synonymous
	870 849	LMR479A_0873			C→A	synonymous
	1 155 986	LMR479A_1182			T→C	synonymous
	1 205 445	LMR479A_1238			C→A	synonymous
	1 315 413	LMR479A_1367			G→A	synonymous
	1 686 793	LMR479A_1730↔ LMR479A_1731		Note: The mutation was located in LMR479A_misc_RNA_30; T-box (RF00230), intergenic region	A→T	RNA/noncoding
	1 822 221	LMR479A_1849			A→C	synonymous
	1 972 976	LMR479A_2001			T→A	synonymous
	2 487 240	LMR479A_2537			G→A	synonymous
	2 504 930	LMR479A_2558			G→T	synonymous
	2 855 890	LMR479A_2909			A→G	synonymous
