# Supplementary material for: Genome Analysis of Listeria monocytogenes Sequence Type 8 Strains Persisting in Salmon and Poultry Processing Environments and Comparison with Related Strains
Source: PLoS One. 2016 Mar 8;11(3):e0151117. doi: 10.1371/journal.pone.0151117 (PMC4783014; doi:10.1371/journal.pone.0151117)
Supplement: S3 Table — Genes in prophages and plasmids are disregarded. (PDF) [file pone.0151117.s003.pdf]

S3 Table: Accessory genome in ST8 isolates relative to *L. monocytogenes* strains EGD-e and 10403S. Genes in prophages and plasmids are disregarded.

	**Locus tag (LMR479A_)**	**Protein length**	**Annotation (R479a GenBank file)**	**Protein family membership (INTERPROSCAN)**	**Domains and repeats (INTERPROSCAN)**
LMR479A_0029-0035 is inserted between lmo0028-lmos01(rli1) and lmo0029 in EGD-e.					
	0029	206 aa	conserved protein of unknown function	None predicted	None predicted
	0030	209 aa	putative ATP-binding cassette protein	None predicted	AAA+ ATPase domain (IPR003593); ABC transporter-like (IPR003439)
	0031	240 aa	conserved membrane protein of unknown function	Protein of unknown function DUF2705 (IPR024295)	None predicted
	0032	237 aa	conserved membrane protein of unknown function	None predicted	None predicted
	0033	254 aa	putative integral inner membrane protein	None predicted	None predicted
	0034	146 aa	conserved exported protein of unknown function	Protein of unknown function DUF2712 (IPR020208)	None predicted
	0035	63 aa	protein of unknown function	None predicted	None predicted
LMR479A_0070-82 is inserted between lmo0061 (homologue to LMR479A_0069) and lmo0072 (homologue to LMR479A_0083) in EGD-e (EGD-e has different genes at this location) LMR479A_0084 is inserted into/in place of (partly) the EGD-e gene lmo0074.<br>lmo0061-0075 are anchor genes for hypervariable hotspot #1 (Kuenne et al., 2013).					
	0070	160 aa	conserved protein of unknown function	None predicted	None predicted
	0071	104 aa	conserved protein of unknown function	None predicted	None predicted
	0072	148 aa	conserved protein of unknown function	None predicted	None predicted
	0073	422 aa	conserved protein of unknown function	None predicted	None predicted
	0074	185 aa	putative secreted lipoprotein	None predicted	None predicted
	0075	151 aa	conserved protein of unknown function	None predicted	None predicted
	0076	141 aa	conserved membrane protein of unknown function	None predicted	None predicted
	0077	75 aa	Predicted protein	None predicted	None predicted
	0083	213 aa	conserved exported protein of unknown function	None predicted	None predicted
	0084	210 aa	conserved protein of unknown function	None predicted	None predicted
LMR479A_0269-0274 is inserted between lmo0257-0258 in EGD-e. LMR479A_0275 is partly present in EGD-e, in unannotated region between lmo0257-0258.					
	0269	331 aa	Transcriptional regulator, LacI family	None predicted	Transcription regulator HTH, LacI (IPR000843), Periplasmic binding protein-like I (IPR028082)
	0270	408 aa	Maltose/maltodextrin transport system substrate-binding protein	None predicted	None predicted
	0271	445 aa	Maltose/maltodextrin transport system permease protein	None predicted	ABC transporter type 1, transmembrane domain MetI-like (IPR000515)
	0272	276 aa	Maltose/maltodextrin transport system permease protein	None predicted	ABC transporter type 1, transmembrane domain MetI-like (IPR000515)
	0273	565 aa	Oligo-1,6-glucosidase	Glycoside hydrolase, family 13 (IPR015902)	Glycosyl hydrolase, family 13, subfamily, catalytic domain (IPR006589), Glycosyl hydrolase, family 13, all-beta (IPR013780)
	0274	480 aa	Sucrose phosphorylase	Sucrose phosphorylase, GftA (IPR022527)	Glycosyl hydrolase, family 13, subfamily, catalytic domain (IPR006589)
	0275	81 aa	conserved protein of unknown function	None predicted	None predicted
LMR479A_0346-0349 is inserted between lmo0334 (homologue to LMR479A_0345) and lmo0340 (homologue to LMR479A_0351) in EGD-e (EGD-e has different genes at this location). LMR479A_0350 is partly present in EGD-e, in unannotated region between lmo0338-0339.					
	0346	131 aa	conserved protein of unknown function	Uncharacterised conserved protein UCP019853 (IPR016767)	None predicted
	0347	39 aa	conserved protein of unknown function	None predicted	None predicted
	0348	76 aa	conserved protein of unknown function	None predicted	Cysteine-rich CPCC domain (IPR025983)
	0349	75 aa	conserved protein of unknown function	None predicted	None predicted
	0350	126 aa	conserved protein of unknown function	None predicted	None predicted

S3 Table, continued:

	Locus tag (LMR479A_)	Protein length	Annotation (R479a GenBank file)	Protein family membership (INTERPROSCAN)	Domains and repeats (INTERPROSCAN)
LMR479A_0528-0532 is inserted between lmo0520 and lmo0521 in EGD-e.					
	0528	1020 aa	Type I restriction enzyme, R subunit	None predicted	Restriction endonuclease, type I, HsdR, N-terminal (IPR007409), P-loop containing nucleoside triphosphate hydrolase (IPR027417), Helicase/UvrB domain (IPR006935), Type I restriction and modification enzyme, subunit R, C-terminal (IPR022625)
	0529	529 aa	Type I restriction enzyme M protein	Restriction endonuclease, type I, HsdM (IPR004546)	S-adenosyl-L-methionine-dependent methyltransferase (IPR029063), N6 adenine-specific DNA methyltransferase, N-terminal domain (IPR022749), S-adenosyl-L-methionine-dependent methyltransferase (IPR029063), DNA methylase, adenine-specific (IPR003356)
	0530	397 aa	conserved protein of unknown function	None predicted	2x Restriction endonuclease, type I, HsdS (IPR000055),
	0531	309 aa	Integrase	None predicted	Integrase/recombinase, N-terminal (IPR023109), Integrase, catalytic (IPR002104)
	0532	358 aa	Type I restriction enzyme, S subunit	None predicted	2x Restriction endonuclease, type I, HsdS (IPR000055),
LMR479A_0811 is inserted in place of lmo0792 (clean change of genes, homologues on either side)					
	0811	421 aa	conserved exported protein of unknown function	None predicted	None predicted
LMR479A_1125-1143 are inserted between lmo1096 (homologue to LMR479A_1124) and lmo1124 (homologue to LMR479A_1144) in EGD-e (EGD-e has different genes at this location, with some short patches of homology). lmo1096-1126 are anchor genes for hypervariable hotspot #8 (Kuenne et al., 2013).					
	1125	637 aa	Site-specific DNA-methyltransferase (Adenine-specific)	N6 adenine-specific DNA methyltransferase, D21 class (IPR002295), Restriction/modification DNA-methylase (IPR001091)	Type III restriction/modification enzyme methylation subunit (IPR022221), 2x S-adenosyl-L-methionine-dependent methyltransferase (IPR029063), DNA methylase N-4/N-6 (IPR002941)
	1126	925 aa	Type III restriction enzyme	None predicted	2x P-loop containing nucleoside triphosphate hydrolase (IPR027417), Helicase/UvrB domain (IPR006935)
	1127	42 aa	Phage integrase family site-specific recombinase (fragment)	None predicted	None predicted
	1128	58 aa	conserved protein of unknown function	None predicted	None predicted
	1131	42 aa	protein of unknown function	None predicted	None predicted
	1132	101 aa	protein of unknown function	None predicted	None predicted
	1133	601 aa	conserved protein of unknown function	Abortive bacteriophage infection, resistance (IPR018891)	None predicted
	1134	225 aa	conserved protein of unknown function	None predicted	Cro/C1-type helix-turn-helix domain (IPR001387)
	1135	233 aa	conserved protein of unknown function	None predicted	Cro/C1-type helix-turn-helix domain (IPR001387)
LMR479A_2136-2138 is inserted between lmo2025-lmos78(rliF) (homologue to LMR479A_2135,rliF unannotated) and lmo2028 (homologue to LMR479A_2139) in EGD-e (EGD-e has different genes at this location). lmo2025-2028 are anchor genes for hypervariable hotspot #9 (Kuenne et al., 2013).					
	2137	151 aa	conserved protein of unknown function	Bacteriophage abortive infection AbiH (IPR025935)	None predicted
	2138	202 aa	conserved protein of unknown function	Bacteriophage abortive infection AbiH (IPR025935)	None predicted
LMR479A_2719-2720 is inserted between lmo2589 (homologue to LMR479A_2718) and a tRNA/rRNA operon (lmor16-18/LMR479A_rRNA4) in EGD-e.					
	2719	215 aa	conserved exported protein of unknown function	None predicted	None predicted
	2720	178 aa	conserved exported protein of unknown function	Peptidase M10A (IPR021190)	Metallopeptidase, catalytic domain (IPR024079), Peptidase M10, metallopeptidase (IPR001818)
LMR479A_2950 is inserted between lmo2812 and lmo2813 in EGD-e.					
	2950	176 aa	conserved protein of unknown function	Protein of unknown function DUF4303 (IPR025409)	None predicted
